# Identifying Patients at Greatest Risk of Mortality due to COVID-19: A New England Perspective

**DOI:** 10.5811/westjem.2020.6.47957

**Published:** 2020-07-08

**Authors:** Aaron A. Smith, Jeremy Fridling, Danyal Ibrahim, Paul S. Porter

**Affiliations:** * Quinnipiac University, Frank H. Netter MD School of Medicine, North Haven, Connecticut; † Trinity Health of New England, Saint Mary’s Hospital, Waterbury, Connecticut

## Abstract

**Introduction:**

Severe acute respiratory syndrome coronavirus 2 (SARS-CoV-2) has spread rapidly since December 2019, resulting in a pandemic that has, as of May 24, 2020, yielded over 5.3 million confirmed cases and over 340,000 deaths.^1^ As businesses move to safely reopen and frontline healthcare workers (HCW) continue to face this crisis, it is essential that health officials know who in the population is at the greatest risk of mortality if hospitalized and, therefore, has the greatest need to protect themselves from being infected. We examined the factors that increase the risk of mortality among hospitalized COVID-19 patients.

**Methods:**

This was a retrospective cohort study including confirmed COVID-19 patients admitted to the four Trinity Health of New England hospitals (THONE) in Connecticut and Massachusetts who either died or were discharged between March 1–April 22, 2020. Demographics, comorbidities, and outcomes of care were extracted from the electronic health record. A model of in-hospital mortality was made using a generalized linear model with binomial distribution and log link.

**Results:**

The analysis included 346 patients: 229 discharged and 117 deceased. The likelihood of in-hospital mortality was increased for patients who were aged 60 or older (relative risk [RR] = 2.873; 95% confidence interval [CI], 1.733–4.764; p = <0.001), had diabetes (RR = 1.432; 95% CI,1.068–1.921; p = 0.016), or had chronic obstructive pulmonary disease (COPD) (RR = 1.410; 95% CI, 1.058–1.878; p = 0.019). Hyperlipidemia had a protective effect, reducing the likelihood of mortality (RR = 0.745; 95% CI, 0.568–0.975; p = 0.032). Sensitivity and specificity of the model were 51.4% and 88.4%, respectively.

**Conclusions:**

Being age 60 or older or having a history of diabetes or COPD are the most useful risk factors associated with mortality in hospitalized COVID-19 patients. As states ease stay-at-home orders, risk factors of severe disease can be used to identify those more likely to have worse outcomes if infected and hospitalized and, therefore, who in particular should continue to follow public health guidelines for avoiding infection: stay home if possible; practice physical distancing; and wear a facemask.

## INTRODUCTION

Severe acute respiratory syndrome coronavirus 2 (SARS-CoV-2) is the causative agent of coronavirus disease 2019 (COVID-19), first described in Wuhan, China, in December 2019. The virus has spread rapidly, resulting in a pandemic that has, as of May 24, 2020, yielded over 5.3 million confirmed cases and over 340,000 deaths.^1^ Overall, the case fatality rate (CFR) is estimated to be around 3.6%.^2^ In hospitals, risk of exposure is high; Saint Mary’s Hospital in Waterbury, CT, saw confirmed SARS-CoV-2 positive cases peak at 68% of the hospital’s census conducted on April 17, 2020, (Paul Porter, MD, phone communication, April 29, 2020). To decrease potential exposures between patients and healthcare workers (HCW), the US Centers for Disease Control and Prevention (CDC) and various specialty societies released guidelines recommending postponement of elective procedures, and many providers turned to telemedicine to conduct their scheduled visits.^3^,^4^,^5^

The risk of death for COVID-19 patients who are hospitalized is significant. In Trinity Health of New England (THONE) hospitals, an internal report released on April 10, 2020, found that 33.2% of hospitalized COVID-19 patients had died since the beginning of the pandemic (Paul Porter, MD, phone communication, April 29, 2020). Emerging studies are aiming to describe characteristics of hospitalized patients and identify risk factors for mortality. Increased age, chronic obstructive pulmonary disease (COPD), cardiovascular disease, diabetes, hypertension, and smoking history are common characteristics that have been observed in hospitalized patients in New York and China.^6^,^7^,^8^ As hospitals respond to this crisis and businesses work to reopen safely, it is crucial to enhance this body of evidence and re-examine the risk factors that increase mortality risk among hospitalized COVID-19 patients. This way, public health officials can identify which individuals carry the greatest risk of death so that precautions may be taken as members of the public consider re-entering the workforce and returning to outpatient offices.

## METHODS

This was a retrospective cohort study of confirmed COVID-19 patients admitted to four THONE hospitals: Saint Mary’s Hospital in Waterbury, CT; Saint Francis Hospital in Hartford, CT; Johnson Memorial Hospital in Stafford, CT; and Mercy Medical Center in Springfield, MA. Expedited, non-exempt institutional review board approval was obtained. The study included patients admitted between March 1–April 22, 2020, who died or were discharged as of April 22, 2020, excluding patients who were still hospitalized. An initial data extraction from the electronic health record system, EPIC, was conducted to acquire demographic information and smoking history. For comorbidities, chart abstraction was conducted following standard abstraction protocol.^9^ Two medical students performed all abstraction using explicit inclusion and exclusion criteria in case selection. Both received the same EPIC training and abstraction guidance, and a sample of 10 patient charts were reviewed simultaneously before proceeding to independent abstraction.

Protocol included checking both the initial internal medicine note after hospital admission and the discharge note, and characterizing a patient as having a comorbidity only if it were either included in their problem list or explicitly stated by their provider in either note. It was at the reviewers’ discretion to determine whether these notes were insufficient; if needed, all other notes post-admission were available for abstraction. Throughout the process, we conducted meetings to ensure abstractors had not encountered problems and were following proper protocol. Monitoring, blinding, and testing of inter-rater agreement were not done.

**Population Health Research Capsule**

What do we already know about this issue?

*Studies from New York and China have shown age, diabetes, chronic obstructive pulmonary disease (COPD), and cardiovascular disease to be associated with COVID-19 mortality*.

What was the research question?

What risk factors are associated with in-hospital mortality among hospitalized COVID-19 patients?

What was the major finding of the study?

*Age 60 or older, diabetes, and COPD increase risk of in-hospital mortality*.

How does this improve population health?

*Awareness of those most at risk of mortality if hospitalized for COVID-19 will focus attention on those who should exercise the greatest caution*.

To test the bivariate relationship between risk factors and in-hospital mortality, we used Fisher’s exact test to compare all categorical variables, while an independent samples T-test was used for the continuous variables. A model of in-hospital mortality was made using a generalized linear model with binomial distribution and log link. We initially included all variables in the model, and we then used a manual, stepwise backward elimination approach to remove non-significant variables.

## RESULTS

The analysis included 346 patients hospitalized for COVID-19: 229 discharged (66.18%), and 117 deceased (33.82%). The study sample had a mean age of 66.86 years and had a high incidence of hypertension (69.7%), hyperlipidemia (48.3%), diabetes (47.1%), cardiovascular disease (46.8%), and neurological disease (27.5%), as seen in Table 1. The deceased population had a significantly higher incidence of hypertension, diabetes, cardiovascular disease, chronic kidney disease (CKD), COPD, and cancer compared to the discharged group. The mean body mass index (BMI) of the study sample was 30.58 kilograms per meter squared, and 45.2% of patients were obese, compared with 39.8% of individuals over age 20 nationally.^10^ The groups also differed significantly with regard to age; the mean age of discharged patients was 63.56, and the mean age of deceased patients was 73.31 (p = <0.001). Applying a Bonferroni correction and using a new p-value cut-off for significance of 0.004, we found that age and COPD remained significant, with diabetes bordering on significance.

The results of the generalized linear model are shown in Table 2. The model showed that the likelihood of mortality was increased for patients who were aged 60 or older (relative risk [RR] = 2.873; 95% confidence interval [CI], 1.733–4.764; p = <0.001), had diabetes (RR = 1.432; 95% CI, 1.068–1.921; p = 0.016), or had COPD (RR = 1.410; 95% C, 1.058–1.878; p = 0.019). Hyperlipidemia had a protective effect, reducing the likelihood of mortality (RR = 0.745; 95% CI, 0.568–0.975; p = 0.032). While hypertension, cardiovascular disease, and cancer were significant in the bivariate analysis, they were insignificant predictors in the model and were therefore removed per our manual, stepwise backward elimination approach. Sensitivity and specificity of the model were 51.4% and 88.4%, respectively.

## DISCUSSION

Our model of COVID-19 hospitalized patients in Connecticut and Massachusetts identified that patients with increased age, diabetes, or COPD were at significantly greater risk of death. An early cohort study of patients in Wuhan, China, also modeled in-hospital mortality, finding advanced age to be a significant predictor, while a meta-analysis of studies on patients across China reported diabetes and COPD as predictors of more severe outcomes.^6^,^7^ A large case series study conducted in the New York City area, while not a predictive model, also showed severe outcomes associated with both age and diabetes.^8^ Specifically, age greater than 65 years and diabetes were associated with a higher incidence of intensive care unit admission and invasive ventilation. While no other single study found age, diabetes, and COPD to be significant predictors of mortality, they all appeared in at least one of these studies. Additionally, similar to our study population, both the New York study and Chinese meta-analyses had high rates of hypertension and diabetes in their overall study populations.

The Chinese meta-analysis identified both hypertension and cardiovascular disease as significant risk factors for mortality. While our study did show both of these characteristics to be significantly more common in deceased patients compared to discharged patients, neither were found by our model to be significant predictors of mortality. The New York study, on the other hand, found no association between cardiovascular disease and severe outcomes. Cancer, while not a predictor of mortality, was also found to be significantly more common in deceased patients compared to discharged patients, which was not seen in other studies. These differences in findings could be due to insufficient sample sizes in this and other studies, variation between sample populations, or variability in the definition of cardiovascular disease. The New York study, for example, used cardiovascular disease as an umbrella term representing hypertension, coronary artery disease, and congestive heart failure and did not comment on the significance of coronary artery disease or congestive heart failure as risk factors. Our population, with a mean age of 66.86, was older than the populations of the studies in Wuhan (56.0) and New York (63), and, with an average BMI of 30.58, it had more obesity than the sample in New York, in which only 41.7% were obese (BMI ≥ 30). Our population also had a higher incidence of hypertension (69.7%), diabetes (47.1%), and COPD (16.8%).

Notably, hyperlipidemia was found to be protective, which differed from the results of other studies.^6^,^7^,^8^ It is possible that this effect can be explained by the outpatient use of statins, as these medications are known to have anti-inflammatory properties; recent studies have even proposed that they could have a role in COVID-19 treatment regimens.^11^,^12^ Our study also differs from the others discussed here, in that it offers a regional perspective; New England is a different geographical and political environment than both New York City and China.

Some regions across the US have seen COVID-19 cases and deaths peak and decline, and many are now seeing a softening of social distancing restrictions. The risk factors that we have identified can be used to aid in the decision-making of HCWs as they guide patients’ impending return to in-person healthcare. Professional society guidelines for physicians’ return to practice are calling for the continued use of telemedicine, when possible, to minimize the exposure for vulnerable or at-risk patients.^13^,^14^ Based on our findings, in which patients with age ≥ 60, diabetes, and COPD were at greater risk of death when infected, we suggest that these risk factors can be used to identify vulnerable patients. HCWs should continue to postpone in-person care for patients with these risk factors. If HCWs themselves have these risk factors, they should protect themselves by continuing to use proper personal protective equipment or postpone in-person care, if possible.

## LIMITATIONS

Chart abstraction yielded several limitations. Some documentation was incomplete or overly brief, likely exacerbated by the overburdened hospital system. This was especially true for those who arrived at the hospital unconscious, obtunded, or otherwise unable to give a complete history. Data gathering was also limited by incomplete adherence to the standards of chart abstraction.^9^ Performance of chart abstractors was not monitored by an external source, abstractors were not blinded to the hypothesis or patient’s group assignment, inter-rater agreement was not tested, and abstraction training was not tested; all are potential sources of bias.

While the final model included only four variables, the modeling process began with 14 variables, yielding 8.36 outcomes per variable. This is less than the ideal number, which increases the likelihood of overfit and type I error. Additionally, we were limited by our small sample size. Low counts of individual comorbidities reduced the likelihood that they would be statistically significant factors in our model.

## CONCLUSION

As governments push to re-open businesses and relax restrictions for those returning to work across all industries, including healthcare, we must apply the same precautions based on identification of risk factors for mortality, which our study identified as patients with age ≥ 60, diabetes, and COPD. Members of the public should continue prevention measures including frequent handwashing, wearing masks, and avoiding close contact. However, individuals with one or more of the identified risk factors should adhere to CDC guidelines and take extra precautions, including maintaining extra distance, disinfecting common surfaces, or staying home if possible while their coworkers return to the office.^15^,^16^ Asymptomatic transmission continues to make the spread of COVID difficult to control; thus, the best way to protect the most vulnerable individuals is to reduce as many potential exposures as possible.^17^,^18^ If we are to reduce burden on the healthcare system and successfully fight this pandemic, we must protect those at greater risk of mortality if hospitalized.

## Figures and Tables

**Table 1:** Bivariate analysis; patient demographics and comorbidities on admission.

Characteristics	Total (n=346)	Discharged (n=229)	Deceased (n=117)	P-value
Mean Age (years)	66.86	63.56	73.31	<0.001*
Mean BMI (kg/m^2^)	30.58	30.94	29.85	0.252
Sex	--	--	--	0.647
Male	194 (56.1%)	126 (55.0%)	68 (58.1%)	--
Female	152 (43.9%)	103 (45%)	49 (41.9%)	--
Ever smoker	140 (44%)	89 (40.3%)	51 (52.6%)	0.05*
Comorbidities	--	--	--	--
Hypertension	241 (69.7%)	151 (65.9%)	90 (76.9%)	0.037*
Hyperlipidemia	167 (48.3%)	107 (46.7)	60 (51.3%)	0.429
Diabetes	163 (47.1%)	95 (41.5%)	68 (58.1%)	0.004*
Cardiovascular disease	162 (46.8%)	96 (41.9%)	66 (56.4%)	0.012*
Neurological disease	95 (27.5%)	59 (25.8%)	36 (30.8%)	0.373
CKD	82 (23.7%)	45 (19.7%)	37 (31.6%)	0.016*
COPD	58 (16.8%)	27 (11.8%)	31 (26.5%)	0.001*
Cancer	51 (14.7%)	27 (11.8%)	24 (20.5%)	0.037*
Asthma	47 (13.6%)	35 (15.3%)	12 (10.3%)	0.246
Hypothyroid	45 (13.0%)	25 (10.9%)	20 (17.1%)	0.128

* meets 0.05 p-value level.

*BMI*, body mass index; *CKD*, chronic kidney disease; *COPD*, chronic obstructive lung disease.

**Table 2:** Generalized linear model; risk factors associated with in-hospital mortality.

Risk factors	RR (95% CI)	P-value
Age of 60 or older	4.70 (2.40–9.12)	<0.001*
COPD	1.41 (1.06–1.88)	0.019*
Diabetes	1.43 (1.07–1.92)	0.016*
Hyperlipidemia	0.75 (0.57–0.98)	0.032*

* meets 0.05 p-value level.

*COPD*, chronic obstructive pulmonary disease; *RR*, Relative Risk; *CI*, confidence interval.

## References

[1] COVID-19 Dashboard by the Center for Systems Science and Engineering (CSSE).

[2] Fu L, Wang B, Yuan T (2020). Clinical characteristics of coronavirus disease 2019 (COVID-19) in China: a systematic review and meta-analysis. J Infect.

[3] Cavallo JJ, Forman HP (2020). The economic impact of the COVID-19 pandemic on radiology practices. Radiology.

[4] COVID-19 Recommendations for management of elective surgical procedures.

[5] Joint Statement: Roadmap for Resuming Elective Surgery after COVID-19 Pandemic.

[6] Wang B, Li R, Lu Z (2020). Does comorbidity increase the risk of patients with COVID-19: evidence from meta-analysis. Aging.

[7] Zhou F, Yu T, Du R (2020). Clinical course and risk factors for mortality of adult inpatients with COVID-19 in Wuhan, China: a retrospective cohort study. Lancet.

[8] Richardson S, Hirsch JS, Narasimhan M (2020). Presenting characteristics, comorbidities, and outcomes among 5700 patients hospitalized with COVID-19 in the New York City area. JAMA.

[9] Gilbert EH, Lowenstein SR, Koziol-Mclain J (1996). Chart reviews in emergency medicine research: Where are the methods?. Ann Emerg Med.

[10] National Center for Health Statistics Obesity and overweight.

[11] Jain MK, Ridker PM (2005). Anti-inflammatory effects of statins: clinical evidence and basic mechanisms. Nat Rev Drug Discov.

[12] Fedson DS, Opal SM, Rordam OM (2020). Hiding in plain sight: an approach to treating patients with severe COVID-19 infection. mBio.

[13] Guidance for Return to Practice for Otolaryngology-Head and Neck Surgery: Part One.

[14] COVID-19 Pandemic Breast Cancer Consortium’s considerations for re-entry.

[15] (2020). COVID-19: Protect yourself. cdc.gov.

[16] (2020). COVID-19: What You Can Do. cdc.gov.

[17] Furukawa NW, Brooks JT, Sobel J (2020). Evidence supporting transmission of severe acute respiratory syndrome coronavirus 2 while presymptomatic or asymptomatic. Emerg Infect Dis.

[18] Gandhi M, Yokoe DS, Havlir DV (2020). Asymptomatic transmission, the Achilles’ heel of current strategies to control Covid-19. New Engl J Med.

